# *Energy per Operation* Optimization for Energy-Harvesting Wearable IoT Devices †

**DOI:** 10.3390/s20030764

**Published:** 2020-01-30

**Authors:** Jaehyun Park, Ganapati Bhat, Anish NK, Cemil S. Geyik, Umit Y. Ogras, Hyung Gyu Lee

**Affiliations:** 1 School of Electrical Engineering, University of Ulsan, Ulsan 44610, Korea; jaehyun@ulsan.ac.kr; 2 School of Electrical, Computer and Energy Engineering, Arizona State University, Tempe, AZ 85281, USA; gmbhat@asu.edu (G.B.); anishnk@asu.edu (A.N.); umit@asu.edu (U.Y.O.); 3 Technology Development, Intel Corporation, Chandler, AZ 85226, USA; cemil.s.geyik@intel.com; 4 School of Computer and Communication Engineering, Daegu University, Gyeongsan-si 38453, Korea

**Keywords:** wearable devices, gesture recognition, energy model, energy harvesting, energy optimization

## Abstract

Wearable internet of things (IoT) devices can enable a variety of biomedical applications, such as gesture recognition, health monitoring, and human activity tracking. Size and weight constraints limit the battery capacity, which leads to frequent charging requirements and user dissatisfaction. Minimizing the energy consumption not only alleviates this problem, but also paves the way for self-powered devices that operate on harvested energy. This paper considers an energy-optimal gesture recognition application that runs on energy-harvesting devices. We first formulate an optimization problem for maximizing the number of recognized gestures when energy budget and accuracy constraints are given. Next, we derive an analytical energy model from the power consumption measurements using a wearable IoT device prototype. Then, we prove that maximizing the number of recognized gestures is equivalent to minimizing the duration of gesture recognition. Finally, we utilize this result to construct an optimization technique that maximizes the number of gestures recognized under the energy budget constraints while satisfying the recognition accuracy requirements. Our extensive evaluations demonstrate that the proposed analytical model is valid for wearable IoT applications, and the optimization approach increases the number of recognized gestures by up to 2.4× compared to a manual optimization.

## 1. Introduction

Designing small form factor wearable devices without degrading user experience can enable pervasive biomedical applications such as gesture-based control, health monitoring, and activity tracking [1,2,3,4]. However, a small form factor generally limits the capacity of the battery, hence requiring frequent battery replacements and charging, which are inconvenient. Lighter flexible batteries have advantages in size and weight, but their capacities (200 mAh @ 1.2 g) [5] are not enough for the seamless operation of wearable devices. Therefore, maximizing the utilization (i.e., useful work) under a tight energy budget is key to the success of wearable IoT devices [6].

**Figure 1:**
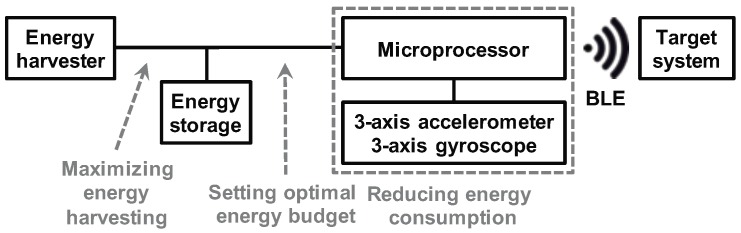
Proposed wearable gesture recognition system. BLE: Bluetooth Low Energy.

Harvesting energy from ambient sources is an attractive way to alleviate the battery problem [7], especially for wearable IoT devices. Among various energy-harvesting resources, it is known that photovoltaic cells (PV-cells) generate 10–100 mW/cm${}^{2}$ [8,9], which can operate the wearable device even without a battery. Researchers have also recently studied other ambient energy sources, most notably thermo-electric [10] and kinetic energy [11,12]. These studies have shown that thermo-electric generators (TEGs) and piezo electric devices can generate up to 50 μW and 2 mW power, respectively. Hence, they are extremely useful and complementary to PV-cells, especially for indoor environments. In this work, we consider the combination of an energy-harvesting source and a small-size energy storage device such as a rechargeable battery or a capacitor, as shown in Figure 1. We complement the energy-harvesting source with a back-up storage device since the amount of harvested energy is intermittent and exhibits significant variations depending on the time and day [13].

The goal of this paper is to maximize the work performed by an energy harvesting wearable device under a given energy budget. To demonstrate the effectiveness of the proposed solution, we employ a gesture recognition application and the system shown in Figure 1. Gesture recognition was chosen as the driver application since it is an important component of biomedical applications, including gesture-based control and interaction with robotic assistive devices. We implemented gesture recognition using a wearable device consisting of an energy-harvesting subsystem, a microprocessor, a 3-axis accelerometer, a 3-axis gyroscope, and a Bluetooth Low Energy (BLE) interface (detailed in Section 3). Hence, our concrete goal in this context becomes maximizing the number of correctly recognized gestures under an energy budget, which is determined by the harvested energy.

This paper makes three novel contributions to address the major challenges in solving the aforementioned optimization goal. First, accurate energy consumption and gesture recognition accuracy models are needed to guide this optimization. Second, the problem should be solved at runtime with minimum implementation overhead. Finally, the optimization methodology has to be validated using an energy-harvesting device and user subject studies to be credible.

Toward this end, we first measured and characterized the power consumption of the sensors, microprocessor, and BLE separately while performing gesture recognition. The detailed energy characterization presented in this paper enabled us to develop a novel compact energy model that can be used at runtime by energy-optimization algorithms. Minimizing energy consumption alone can degrade accuracy and reduce the number of correctly recognized gestures. Therefore, it was also necessary to constrain the minimum allowed recognition accuracy. To achieve this, we analyzed the recognition accuracy as a function of the gesture recognition duration by performing user studies. The models presented in this paper are currently the most detailed and accurate energy consumption and recognition accuracy characterizations available, to the best of our knowledge. Finally, we design a novel computationally efficient algorithm using these models to maximize the number of recognized gestures at runtime under the energy budget and accuracy constraints. Our extensive experimental evaluations demonstrate that the proposed approach increases the number of recognized gestures by up to 2.4× compared to a manual optimization while consuming one order of magnitude less energy compared to the state-of-the-art approaches based on radar [14] and electromyography ()EMG) [15] processing.

In summary, the novel contributions of this paper are as follows:

- A detailed energy consumption analysis for wearable gesture recognition devices and novel analytical models considering different operating voltage levels;
- An algorithm to maximize the number of recognized gestures under the given energy budget and accuracy constraints;
- Empirical evaluations using a wearable device prototype, which demonstrate up to 2.4× increase in the number of recognized gestures compared to a manual optimization;

The rest of this paper is organized as follows: we review the related work in Section 2. We present the system overview and the proposed algorithm in Section 3 and Section 4. We discuss the experimental results in Section 5 and summarize the conclusions in Section 6.

## 2. Related Work

Wearable IoT devices have been studied extensively due to their form factor and cost benefits. Researchers have proposed sensor networks, gesture-based control, health monitoring, and activity monitoring as potential applications of IoT devices [16,17,18,19,20,21,22]. Gesture recognition using wearable devices has received significant attention due to its applications in human-computer interaction, gesture-based control, and virtual reality [23,24,25,26]. For instance, impedance sensing [25] and EMG sensors [23,26] on wearable devices are used to recognize gestures. The use of motion sensors with dynamic time warping (DTW) [24,27] and template matching methods [27] have also been proposed to identify gestures. While these studies achieved high recognition accuracy, most of them were implemented and tested offline on the host machines. In contrast, we propose a low-power implementation of gesture recognition on a wearable prototype under the given energy budgets considering energy-harvesting applications.

Wearable devices need to operate under tight energy budgets due to their small battery capacities. Therefore, a significant amount of research has focused on wearable devices with energy harvesting [7,28,29]. As examples, a jacket with solar and thermal energy harvesting [7] and a multi-sensor wearable bracelet with body-heat harvesting [28] have been proposed. Energy harvesting in wearable IoT devices also requires energy management and energy allocation algorithms [30,31,32]. In this regard, the work in [29] manages sleep and wake-up cycles to enable 24-h operation of the wearable sensor node whereas the work in [30] allocates the duty cycle of a wireless sensor node for every control interval. Similarly, a dynamic programming approach was used to perform near-optimal energy allocation for self-powered wearable devices [32]. Unlike prior approaches, our work assumes that the energy budget for each time horizon is provided by a similar algorithm and maximizes the number of gestures recognized under this energy budget.

In addition to the proposed energy management, low-power computing is critical for wearable devices due to the limited energy budget. Recent research has focused on the accuracy–power trade-off in wearable devices [33,34,35,36]. For instance, the technique presented in [33] used dynamic sensor selection to minimize the power consumption of a gesture recognition body area network. This maximized the network lifetime. The work in [34] proposed an algorithm to perform optimal feature selection in wearable sensor networks. In contrast to these approaches, we propose a novel runtime algorithm that maximizes the number of gestures that can be recognized in a given time horizon. While our previous work in [37] formulated the problem as a nonlinear optimization problem and proposed a graphical solution, in this work we first present our detailed characterization of the energy consumption in wearable devices. Then, we provide a theoretical optimization methodology to maximize the number of gestures recognized and validate the proposed methodology with experimental measurements.

## 3. Target System Overview

### 3.1. Energy-Harvesting Wearable Device Prototype

**Figure 2:**
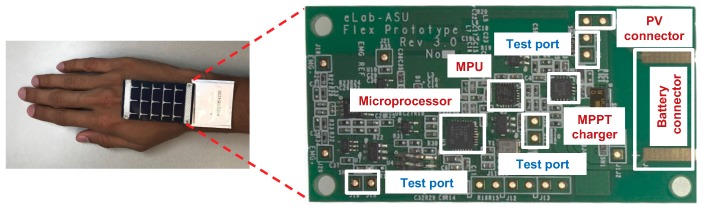
Gesture recognition prototype. MPPT: maximum power point tracking; MPU: motion processing unit; PV: photovoltaic.

We designed a wearable gesture recognition prototype which integrates a PV module and a maximum power point tracking (MPPT) charger as the energy harvesting system, as shown in Figure 2. The MPPT charger (TI BQ25504 [38]) is connected to the output of the PV module. It runs an MPPT algorithm to maximize the power provided to the energy storage, which is a lithium polymer battery in our case. The MPPT algorithm ensures that the maximum power is transferred from the PV-cell to the energy storage device regardless of the changes in the load current.

The dimensions of the PV module used in this work (SP3-37) [39] are 37 mm × 64 mm. It generates 66 mW power, which amounts to a power density of 2.8 mW/cm${}^{2}$. This is smaller than typical rigid modules since the SP3-37 is a physically flexible PV module with lower performance and it loses some white space due to electrical connections. Finally, we use a lithium polymer battery (DMI PGEB0054338) [5] which weighs 1 g with 45 mAh capacity for energy storage to alleviate the problem caused by the non-negligible energy fluctuation over a day.

A motion processing unit (InvenSense MPU-9250) [40] collects the user motion data while the microprocessor (TI CC2650) [41] executes a gesture classifier. The microprocessor also has the ability to perform BLE communication. In the prototype, we also added test ports to measure the power consumption of each major component. As shown in Figure 2, our prototype is designed and implemented to be attached on the hand.

The amount of harvested energy determines the energy budget that can be exploited by the device. To be practical, this system has to maximize the number of intended operations (in our case, gesture recognition) under this budget, while maintaining a minimum level of recognition accuracy. Therefore, we present a methodology to maximize the number of recognized gestures with a given energy budget and accuracy constraint.

### 3.2. Problem Formulation

Given the characteristics of the energy-harvesting system, one can determine the energy that can be harvested over a finite horizon $t_{h}$ [32]. We use this amount as the energy budget $E_{b}$ available for the wearable device during the time $t_{h}$. The gesture recognition duration $t_{g}$ is defined as the time spent by the device to infer a single gesture, as summarized in Table 1. The wearable device actively senses the hand motion and processes the data during this period, which takes a portion of $t_{h}$. We denote the number of gestures recognized within the finite horizon by $N_{g}\left(t_{g}\right)$, since it is a function of the gesture recognition duration. The energy consumption per gesture $E_{g}\left(t_{g}\right)$ is a function of $t_{g}$, because $t_{g}$ determines the active time of the processor and sensor. Similarly, the energy consumption of the device during the idle time is denoted by $E_{i}\left(t_{g}\right)$. Finally, the energy consumed for transmitting the recognized gesture is denoted by $E_{comm}$. With this notation, the proposed optimization problem is formulated as:

(1) $$\begin{array}{cc} \mathrm{maximize}\;N_{g}\left(t_{g}\right)\;\mathrm{such}\;\mathrm{that} & \end{array}$$

(2) $$\begin{array}{ccc} E_{total}\left(t_{g}\right)=E_{g}\left(t_{g}\right)\cdot N_{g}\left(t_{g}\right)+E_{i}\left(t_{g}\right)+E_{comm} & \leq & E_{b} \end{array}$$

(3) $$\begin{array}{ccc} G_{acc}\left(t_{g}\right) & \geq & G_{acc,min} \end{array}$$

The first constraint in this formulation ensures that the total system energy consumption is always less than the energy budget. The second constraint guarantees that the accuracy of the gesture recognition $G_{acc}\left(t_{g}\right)$ is greater than a minimum accuracy $G_{acc,min}$. Note that $G_{acc}\left(t_{g}\right)$ is a function of $t_{g}$, since $t_{g}$ determines the number of data points used for gesture recognition given the sampling frequency.

Solving the optimization problem given by Equations (1)–(3) at runtime is not easy since both the objective and constraints are nonlinear. Moreover, system dependencies make it hard to model the behavior of $E_{g}\left(t_{g}\right)$ and $E_{i}\left(t_{g}\right)$.

### 3.3. Overview of the Proposed Approach

**Figure 3:**
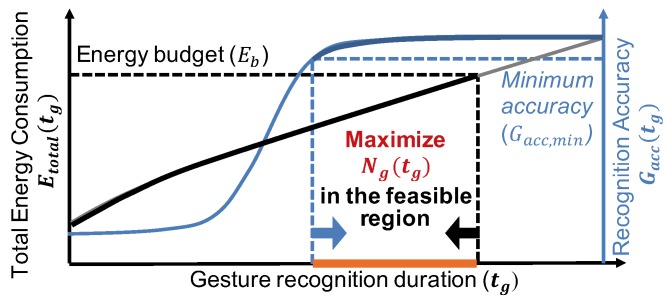
Energy budget and minimum accuracy requirements constrain the gesture recognition duration $t_{g}$ from above and below, respectively. Hence, we maximize the number of recognized gestures within the feasible region.

The energy consumed per gesture is an increasing function of the gesture recognition duration $t_{g}$ since a longer duration increases the active time of the sensors and processor. While precise characterization requires a detailed model as developed in Section 4.1, it can be conceptually illustrated by the left axis in Figure 3. Hence, the gesture recognition duration $t_{g}$ is bounded from above by the given energy budget $E_{b}$. Similarly, the gesture recognition accuracy is expected to improve when a larger number data samples and longer processing time is used. Again, its precise behavior can be found only after user studies, but we can conceptualize it as a non-decreasing function of the gesture recognition duration, as illustrated by the right axis in Figure 3. Consequently, a minimum accuracy requirement bounds the gesture recognition duration $t_{g}$ from below, regardless of the shape of the curve. As a result, the feasible region for the optimization problem is the intersection of the regions for energy and accuracy, as highlighted in Figure 3.

To quantify a solution within the feasible region, we need to express the total energy consumption as a function of the gesture recognition duration, that is, $E_{g}\left(t_{g}\right)$ and $E_{i}\left(t_{g}\right)$ should be derived. Then, we need to model $N_{g}\left(t_{g}\right)$ such that it can be maximized within the feasible region. We solved this optimization problem through following steps:

1. Develop the gesture recognition algorithm on the target hardware and characterize the power consumption of individual components (Section 4.1 and Section 4.2);
2. Construct mathematical energy consumption models using this characterization (Section 4.3);
3. Derive an expression for $N_{g}\left(t_{g}\right)$ and its maximum point using the mathematical models (Section 4.4);
4. Combine the output of step 3 with the lower bound on $t_{g}$ given by the gesture recognition accuracy $G_{acc,min}$ to find the optimal solution. Note that we characterize $G_{acc}\left(t_{g}\right)$ through user studies presented in Section 5.4.

## 4. Energy-Optimal Gesture Recognition

### 4.1. Gesture Recognition Algorithm

**Figure 4:**
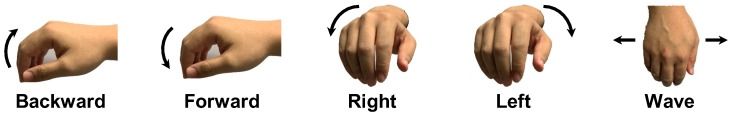
Illustration of the target gestures.

We define five gestures made by one hand (i.e., backward, forward, left, right, and wave), as shown in Figure 4. In addition, we include a stationary gesture to detect when the device is inactive.

The target gestures can be classified using a variety of supervised learning algorithms, such as support vector machine (SVM), decision tree, logistic regression, and neural network (NN). Selecting the appropriate algorithm depends on the input data size, accuracy, and latency requirements, as well as available computational power and memory. In our application, the input is provided by a 3-axis accelerometer with 50 Hz sampling rate. Since common gestures take approximately 0.8 s [42], a baseline implementation with $t_{g}=$ 0.8 s leads to 3× 50 Hz ×0.8 s = 120 input features. We aim at a flexible solution that can be easily extended to have more number of gestures and input features. Our goal is to achieve 90% or higher accuracy on a small wearable IoT device. While both SVM and NN implementations meet the accuracy requirement on our test data, we adopt an NN due to its flexibility. We performed a thorough design space exploration and designed an NN with a single hidden layer with four neurons. The details of this design space exploration are presented under experimental results in Section 5.2 because it does not affect the proposed energy algorithm.

We employ two versions of the NN for the gesture recognition application:

- **Baseline NN** uses all 120 accelerometer samples collected by the three-axis accelerometer during $t_{g}$ as input features.
- **Reduced NN** employs transformed features derived from the raw accelerometer data. We utilize the minimum, maximum, and mean values of each axis (x,y,z) over $t_{g}$. Hence, these amounts to a total of nine input features. Since the number of transformed features does not depend on $t_{g}$, we can change it at runtime.

### 4.2. Operation and Energy Measurements

**Figure 5:**
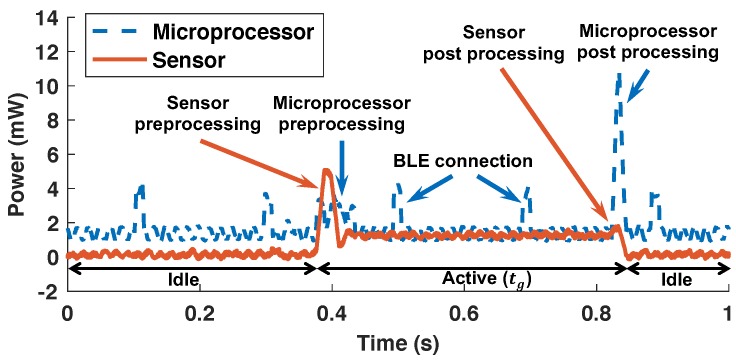
Power consumption during a gesture recognition when $t_{g}=400$ ms.

Figure 5 shows the power consumption of the microprocessor and the sensor (i.e., accelerometer) for a single gesture. The power consumption of the microprocessor is presented using a dashed blue line, while the sensor power consumption is presented using a solid red line. The default behavior of the target device is to stay in the idle state, as shown on the left side of Figure 5. The power consumption of the sensor is close to zero at this state, while the microprocessor consumes about 1.3 mW of power even in the idle state. When the user initiates a gesture, the accelerometer senses the movement and wakes the system up. The first step after system wake-up is to perform a pre-processing routine that prepares the accelerometer and microprocessor for the gesture recognition. Next, the accelerometer samples motion data for a duration of $t_{g}$ while the user is performing the gesture. The power consumption of the sensor increases by about 1.2 mW when sampling the motion. Once the data acquisition is complete, the sensor goes back to the idle state after transmitting data to the microprocessor. In parallel, the microprocessor extracts the features and identifies the gesture using the NN, which consumes significant power as shown in Figure 5 using the microprocessor post-processing annotation. The power consumption of the microprocessor when performing the gesture processing is about 10 mW at peak. Finally, the microprocessor transmits the recognized and classified gesture to the host using the BLE protocol, which consumes about 4 mW of power. We also see periodic peaks in the microprocessor power consumption that are necessary to maintain an BLE connection active.

### 4.3. Energy Consumption Modeling

**Figure 6:**
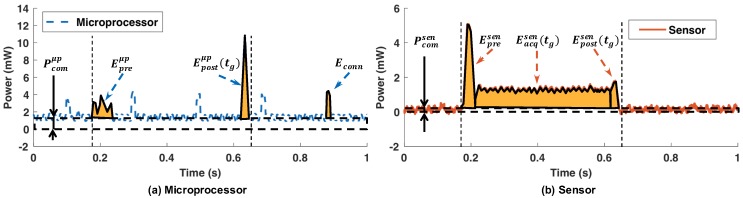
Components of active state energy when $t_{g}=400$ ms.

The power measurements shown in Figure 5 provide useful insights, but cannot be directly used to solve our optimization problem. Hence, we model the energy behavior of the gesture recognition system based on the results of power consumption measurements. Figure 6 shows the detailed power consumption behaviors and corresponding energy models for the microprocessor and sensor, separately.

**Active state energy:** The active energy consumption per gesture $E_{g}\left(t_{g}\right)$ consists of the energy consumption of the microprocessor, $E_{act}^{\mu p}\left(t_{g}\right)$, and of the sensor, $E_{act}^{sen}\left(t_{g}\right)$, in active states, as illustrated in Figure 6a. Hence, we can express it as:

(4) $$E_{g}\left(t_{g}\right)=E_{act}^{\mu p}\left(t_{g}\right)+E_{act}^{sen}\left(t_{g}\right)$$

$E_{act}^{\mu p}\left(t_{g}\right)$ can be modeled by adding the peak components to the common static energy consumption as follows:

(5) $$E_{act}^{\mu p}\left(t_{g}\right)=P_{com}^{\mu p}\cdot t_{g}+E_{pre}^{\mu p}+E_{post}^{\mu p}\left(t_{g}\right)$$

where $P_{com}^{\mu p}$, $E_{pre}^{\mu p}$, and $E_{post}^{\mu p}$ are the microprocessor’s common static power consumption, preprocessing energy consumption, and post-processing energy consumption, respectively. Note that the energy consumption of preprocessing does not depend on $t_{g}$.

Similarly, $E_{act}^{sen}\left(t_{g}\right)$ can be decomposed as illustrated in Figure 6b. Hence, it can be written as:

(6) $$E_{act}^{sen}\left(t_{g}\right)=P_{com}^{sen}\cdot t_{g}+E_{pre}^{sen}+E_{acq}^{sen}\left(t_{g}\right)+E_{post}^{sen}\left(t_{g}\right)$$

where $P_{com}^{sen}$, $E_{pre}^{sen}$, $E_{acq}^{sen}\left(t_{g}\right)$, and $E_{post}^{sen}\left(t_{g}\right)$ are the sensor’s common static power consumption, the preprocessing energy consumption, the data acquisition energy consumption, and the post-processing energy consumption in the sensor, respectively.

**Idle state energy:** The energy consumption of the system during the idle state is described as follows:

(7) $$\begin{array}{c} E_{i}\left(t_{g}\right)=E_{idle}^{\mu p}\left(t_{g}\right)+E_{idle}^{sen}\left(t_{g}\right) \end{array}$$

where $E_{idle}^{\mu p}\left(t_{g}\right)$ and $E_{idle}^{sen}\left(t_{g}\right)$ are the total energy consumption of the microprocessor and the sensor in idle state, respectively. The idle time of the system can be calculated by subtracting the total active time from $t_{h}$. Then, $E_{idle}^{\mu p}\left(t_{g}\right)$ can be modeled as below:

(8) $$E_{idle}^{\mu p}\left(t_{g}\right)=P_{com}^{\mu p}\left(t_{h}-t_{g}\cdot N_{g}\left(t_{g}\right)\right)$$

Similarly, the sensor does not have any operation during the idle state. Hence, $E_{idle}^{sen}\left(t_{g}\right)$ can be written as:

(9) $$E_{idle}^{sen}\left(t_{g}\right)=P_{com}^{sen}\left(t_{h}-t_{g}\cdot N_{g}\left(t_{g}\right)\right)$$

**Communication energy:** Since the BLE communication uses a fixed time interval $t_{conn}$ to maintain the connectivity, the wearable system uses the upcoming slot to transmit the data. Hence, the energy consumption caused by BLE communication $E_{comm}$ during the time horizon $t_{h}$ can be described as follows:

(10) $$E_{comm}=t_{h}/t_{conn}\cdot E_{conn}$$

where $E_{conn}$ is the energy consumption of BLE packet exchange in each time period. Note that $E_{conn}$ is the additional energy consumption due to BLE communication. Hence, we have to consider the common static energy consumption when we calculate the energy per bit transmission.

We use the measured energy consumption values to obtain the constant terms in the energy models of the microprocessor and the sensor. Using these values, the energy models are expressed as a function of $t_{g}$. Detailed validation of the energy model is presented in Section 5.3, while the numeric values are summarized in Table 2.

### 4.4. The Proposed Optimization Methodology

The optimization goal in this work is to maximize $N_{g}\left(t_{g}\right)$. Therefore, we start with expressing $N_{g}\left(t_{g}\right)$ as a function of processor and sensor energy consumption. From Equations (1) and (2), we can express $N_{g}\left(t_{g}\right)$ as:

(11) $$N_{g}\left(t_{g}\right)\leq \frac{E_{b}-E_{comm}-E_{i}\left(t_{g}\right)}{E_{g}\left(t_{g}\right)}$$

By substituting $E_{i}\left(t_{g}\right)$, $E_{comm}$, and $E_{g}\left(t_{g}\right)$ using Equations (5)–(6) and Equations (8)–(10), we can re-write Equation (11) as:

(12) $$N_{g}\left(t_{g}\right)\leq \frac{E_{b}-t_{h}/t_{conn}\cdot E_{conn}-\left(P_{com}^{\mu p}+P_{com}^{sen}\right)\cdot t_{h}}{E_{pre}^{\mu p}+E_{post}^{\mu p}\left(t_{g}\right)+E_{pre}^{sen}+E_{acq}^{sen}\left(t_{g}\right)+E_{post}^{sen}\left(t_{g}\right)}$$

The numerator of Equation (12) represents the energy budget for gesture recognition, which is fixed for each finite horizon, $t_{h}$. It is evaluated by subtracting BLE energy consumption and idle energy consumption, as we have to spend this energy at a minimum to keep the system running. Note that time parameters $t_{h}$ and $t_{conn}$ in the numerator are constant. In addition, the energy and power parameters $E_{b}$, $E_{conn}$, $P_{com}^{up}$, and $P_{com}^{sen}$ in the numerator are also independent of $t_{g}$. Therefore, we conclude that the numerator is independent of $t_{g}$ and it does not change during each finite time horizon. Finally, we can prove that the numerator of Equation (12) is nonnegative [43].

The denominator of Equation (12) represents the sum of the dynamic energy consumption for one gesture recognition. This means that the number of gestures $N_{g}\left(t_{g}\right)$ is maximized when we minimize the dynamic energy consumption of one gesture recognition. $E_{acq}^{sen}\left(t_{g}\right)$, $E_{post}^{\mu p}\left(t_{g}\right)$, and $E_{post}^{sen}\left(t_{g}\right)$ are increasing functions of $t_{g}$, while the remaining two terms are independent of $t_{g}$. That is, the denominator is an increasing function of $t_{g}$. Consequently, we can show that maximizing the number of recognized gestures $N_{g}\left(t_{g}\right)$ is equivalent to minimizing $t_{g}$ [43]. As specified in Equation (3), $t_{g}$ is bounded from below by the accuracy constraint $G_{acc,min}$. Therefore, the optimization problem is solved by choosing the minimum $t_{g}$ that meets the accuracy constraint.

## 5. Experimental Evaluation

### 5.1. Experimental Setup

**Power consumption measurements:** We designed and implemented the custom wearable prototype shown in Section 2 for our experiments. In order to measure the power consumption of the microprocessor and accelerometer separately, we added test points to the prototype. With these test points, we profiled the power consumption of the microprocessor and accelerometer using an NI PXIe-6356 DAQ system [44]. In the experiments, we sampled the power consumption with a 5 kHz frequency to capture the power consumption profiles at a fine-grained level.

**User studies:** We performed user studies to validate the proposed optimization algorithm. To this end, we first obtained data from seven users while performing the target gestures. For each gesture performed by each user, the wearable device first sampled the accelerometer and used the NN to identify the gesture. Then, the identified gesture was transmitted to a host device, such as a smartphone or a laptop. In the data collection phase of the study, we also transmitted the raw acceleration data to the host such that a classifier could be trained. With this protocol, we obtained a total of 30 datasets, each containing 50 gestures. Of these 30 datasets, we reserved 10 sets for the NN training. Following popular machine learning flows, we reserved 80% of the data for training, 10% for cross-validation, and 10% for testing. Finally, the 20 remaining datasets were used to test the accuracy of the NN. This data was never seen by the NN so that the robustness of the network could be evaluated fairly.

### 5.2. Neural Network Classifier Design

**Figure 7:**
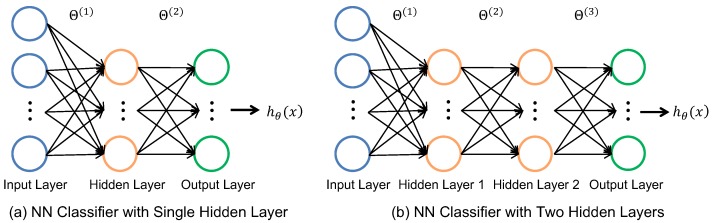
Neural network (NN) classifier architectures used in the design space explorations.

The classifier should achieve the recognition accuracy target while minimizing the energy consumption and area (i.e., the number of weights in this context). To enable an efficient design space exploration, we implemented a programmable NN classifier that allows the number of hidden layers and neurons to be changed. Figure 7 shows the structure of the NN classifier with one and two hidden layers, respectively. We start with the input layer that takes the input features for the current gesture. This amounts to a total of 120 features in the case of the baseline NN and 9 features for the reduced NN. After the input layer, we included either one or two hidden layers for the design space exploration. The neurons in the hidden layer use the sigmoid activation function to introduce non-linearity in the NN classifier. The output of the hidden layers feeds the output layer neurons. The output layer consists of six neurons—one neuron for each gesture and a neuron for the stationary gesture such that we can identify when the user’s hand is stationary. Neurons in this layer also include the sigmoid activation function to generate the probabilities of each gesture. We chose the gesture with the highest output probability as the final gesture.

After choosing these two network structures, we performed a design space exploration by varying the number of neurons in the hidden layers. We trained each NN classifier instance and obtained the gesture recognition accuracy. During these experiments, the number of training epochs was set to 300, while the batch size was 50. We chose these values since they offer a good trade-off between training time and accuracy. Furthermore, we performed a five-fold cross-validation training to ensure robustness.

**Figure 8:**
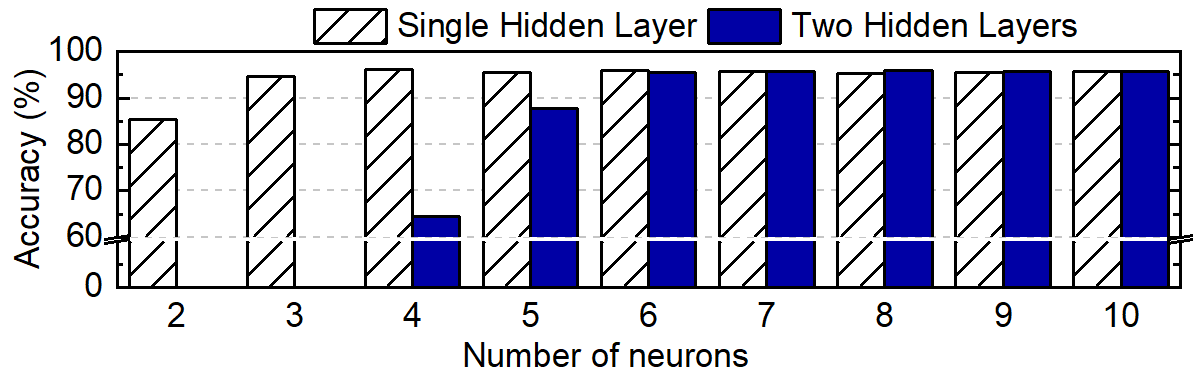
Recognition accuracy according to the number of neurons in the hidden layer.

Figure 8 plots the accuracy of the gesture recognition as a function of the number of neurons in the hidden layers. The *x*-axis in the figure represents the total number of neurons used in the hidden layers. The accuracy values shown in the figure correspond to the median accuracy obtained when using five-fold cross-validation. We used the median accuracy to ensure that the neural network was robust in the five-fold cross-validation. We observed that the median accuracy of a single hidden layer network with two neurons was only about 85%. Furthermore, it exhibited a high variance in accuracy for different folds. The median accuracy increased up to four neurons and then saturated at around 96%. We observed a similar trend in accuracy for two hidden layers: the accuracy saturated at 96% once the total number of neurons was seven. Since our goal was to obtain accuracy greater than 90% while keeping the memory footprint small, we chose to use a single-hidden-layer network with four neurons in the hidden layer. After choosing the neural network structure, we performed the final training of the chosen NN classifier. We obtained 96.5%, 97.4%, and 98.4% accuracy for the training, cross-validation, and testing data, respectively.

### 5.3. Energy Model Validation

We validated the energy consumption models presented in Section 4.3 by running the gesture recognition application with the baseline and reduced NNs. The gesture recognition duration $t_{g}$ of the baseline NN was set to 800 ms because $t_{g}$ cannot be changed at runtime. The gesture recognition duration $t_{g}$ in the reduced NN was swept from 400 to 800 ms in increments of 100 ms.

The extracted values of the key model parameters are summarized in Table 2. These values were extracted by fitting the measurement results to the proposed energy models. We also report parameters for three different supply voltages because the battery voltage varied with the status of the energy-harvesting and discharging operation to the target device. In addition, the supply voltage impacted both the device power consumption and the number of recognized gestures under a given energy budget. In our experiment, the supply voltage could be considered to be constant within one gesture recognition interval because voltage changes were relatively slow compared with the gesture recognition interval.

The proposed models achieved a mean percentage error of only 0.01% for the baseline NN. The corresponding error for the reduced NN ranged from 0.01% to 0.12%. The maximum error across all data points was only 2.9%. This shows that the proposed models can be used for energy optimization.

### 5.4. Gesture Recognition Accuracy Analysis

**Figure 9:**
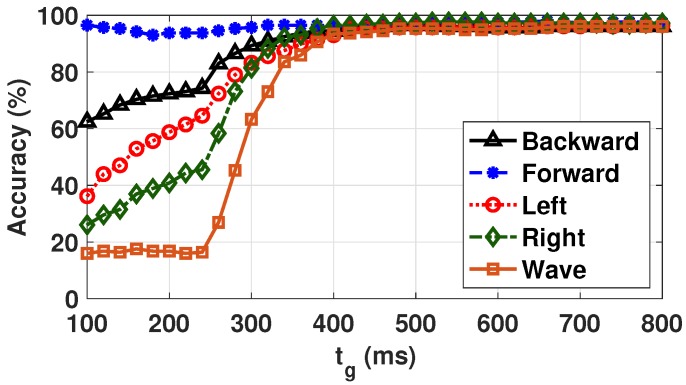
Accuracy of gesture recognition with the reduced NN for all users.

We used gesture recognition experiments from the seven users to evaluate the accuracy of the proposed NN classifier. We provided a random sequence of 50 gestures for the user to perform. The NN classified the gesture and transmitted it to the host device, which stored it for the accuracy analysis. We repeated the experiment three times for each user to obtain a total of 150 gestures per user. After completing the experiments, we compared the classification output of the NN and the reference gesture to obtain the accuracy. By performing an offline analysis using the raw acceleration data with $t_{g}$ = 800 ms, we also obtained the accuracies for multiple values of $t_{g}$, from 100 to 800 ms. The same collected data was used to avoid the overhead of data collection for each value of $t_{g}$. Figure 9 shows the accuracy of the NN as a function of the gesture recognition duration $t_{g}$. We observed that the accuracy of all gestures was greater than 90% when $t_{g}$ > 380 ms. The accuracy degraded rapidly when $t_{g}$ was reduced below 380 ms. We observed that a lower value of $t_{g}$ made it harder to distinguish the features of each gesture. Furthermore, a gesture may not even be completed in less than 380 ms. For instance, the NN needed a larger number of samples to extract the features of the wave gesture, leading to the rapid degradation of its accuracy. By taking into account the accuracy change with $t_{g}$, we chose 380 ms as the lower bound for $t_{g}$. We also observed that the accuracy of the baseline NN degraded faster with lower $t_{g}$, since it used raw acceleration samples as features. Nevertheless, our aim was to maintain accuracy greater than 90% for the baseline NN as well.

### 5.5. Optimization Results

Based on the results of Figure 9 and Section 4.4, we confirmed that the minimum $t_{g}$ satisfying the accuracy requirement could maximize the number of gestures recognized by the wearable devices during $t_{h}$. In the experiment, we set $t_{h}$ to one minute considering the length of a single gesture and the characteristics of energy harvesting which fluctuate according to environmental conditions. Three energy budgets, $E_{b}$ = {120 mJ, 180 mJ, 240 mJ} were considered to evaluate the proposed optimization methodology. Each energy budget, from the first, corresponded to the harvested energy during time $t_{h}$ when the harvested power from the ambient was equal to 2 mW, 3 mW, and 4 mW, respectively. We also considered three different voltage levels (2.7, 3.0, and 3.3 V) of the energy storage to show that the proposed optimization algorithm maximized the number of recognized gesture regardless of the voltage level of the energy storage.

We evaluated the effectiveness of the proposed methodology by comparing the number of recognized gestures to the results of the baseline NN as well as the manually optimized version of the baseline NN by increasing $t_{conn}$. Our solution (labeled as *Reduced*) used the same $t_{conn}$ as the manually optimized baseline to present the benefit of the algorithm excluding the effect of $t_{conn}$ change. The minimum accuracy of gesture recognition was set to 90% throughout the experiments.

**Figure 10:**
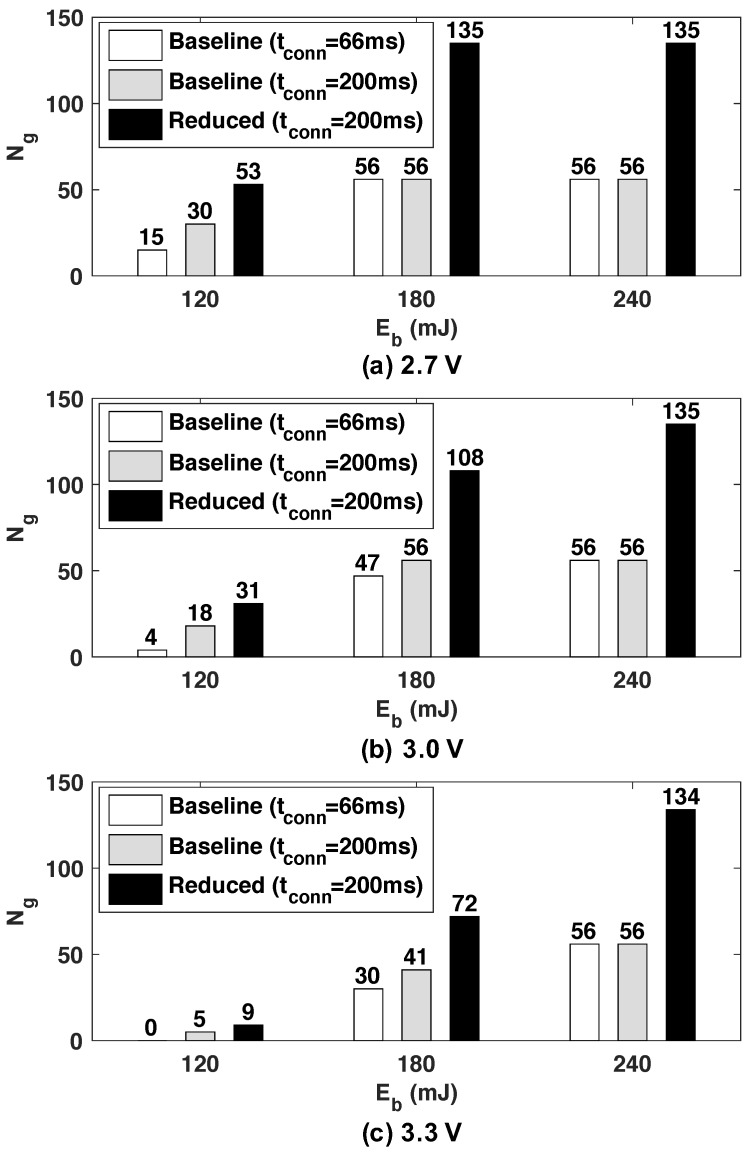
Comparison of the number of recognized gestures for various energy budgets and energy storage voltages.

Figure 10 shows the number of gestures recognized by the three versions of the gesture recognition classifier. When the energy budget was set to 120 mJ, the baseline NN was able to recognize 15 or fewer gestures during time $t_{h}$ depending on the voltage level of the energy storage. At 3.0 V, only 4.6% of the energy budget was used for recognizing four gestures, while the static energy and BLE communication consumed 71.3% and 24.1% of the energy budget, respectively. If the level of supply voltage decreased to 2.7 V, the number of recognized gestures increased to 15 because the wearable device consumed less common static power at 2.7 V. Conversely, when the voltage level was increased to 3.3 V, the wearable device was unable to recognize any gestures since the energy budget was not sufficient for even the static and communication energy. The baseline method with longer $t_{conn}$ recognized more gestures—from 5 to 30—by reducing BLE communication energy. Finally, the proposed optimization recognized 9 to 53 gestures which represented an improvement of 1.7× to 1.8× at all supply voltages, compared to the manually optimized baseline.

We also analyzed the effect of energy budget changes. Since the increased energy budget makes the portion of the energy consumed by BLE connection and static energy decrease significantly, a greater portion of energy can be used to recognize the more gestures. As shown in the figure, all three versions recognized more gestures than the lower energy budget. In particular, we observed significant improvements when the supply voltage was 3.3 V because the device consumed higher power at the higher supply voltage and the energy budget used for the recognition was increased more than in the low supply voltage. Overall, our optimization approach utilized the increased budget more efficiently than the baselines, with 1.8× to 2.4× enhancement over the manually optimized baseline NN. Similarly, the proposed approach consumed 1.3 mW ∼ 4.3 mW while recognizing a gesture. This is one order of magnitude lower compared to the state-of-the-art approaches based on radar [14] and EMG [15] processing, respectively. We observed that when the energy budget was 240 mJ, the maximum number of gestures that could be recognized by our approach and the optimized baseline were not limited by the energy budget, but by the time.

**Figure 11:**
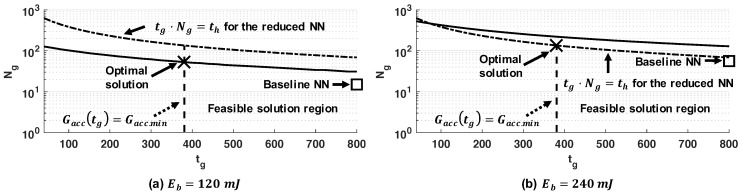
Illustration of the optimal solution for different energy budgets when the energy storage voltage is 2.7 V.

Figure 11 illustrates the optimization results in more detail. For simplicity, we only provide the results with a 2.7 V supply voltage. The dotted curve denotes the implicit upper bound induced by $t_{h}$ while the vertical dashed line indicates the accuracy constraint. The result of baseline NN is presented just with the □ marker because the baseline NN was not able to adopt a $t_{g}$ change at runtime. The results of our approach are represented by the solid curve varied the number of gestures $N_{g}\left(t_{g}\right)$.

As shown in Figure 11, $N_{g}\left(t_{g}\right)$ is a decreasing function of $t_{g}$. Hence, we concluded that the minimum gesture recognition duration satisfying the accuracy requirement determined the optimal operating point, as stated in Section 4.4.

When the energy budget was increased to 240 mJ, the $N_{g}\left(t_{g}\right)$ curve shifted up, as shown in Figure 11b. This meant a larger number of recognized gestures, as expected. We observed that $N_{g}\left(t_{g}\right)$ started intersecting the timing constraint given by the dashed curve. As a result, the constraint due to time horizon ($t_{g}\cdot N_{g}\left(t_{g}\right)\leq t_{h}$) determined the maximum number of gestures. Hence, the optimal point was at the corner of the feasible region.

## 6. Conclusions

Biomedical applications are becoming popular with the advances in wearable IoT devices. Despite their significant potential, the useful lifetime of wearable devices is critically limited due to limited battery capacity (hence, energy). This paper addressed this problem by proposing a novel optimization algorithm for energy-harvesting wearable devices. We first formulated an optimization problem to maximize the number of recognized gestures under tight energy budget and accuracy constraints. To solve this problem, we constructed compact analytical energy consumption models and gesture recognition accuracy characterizations by performing experiments using a wearable device prototype. Finally, we proved that maximizing the number of recognized gestures is equivalent to minimizing the gesture recognition duration from the analytical model.

The proposed technique was demonstrated using a gesture recognition prototype. Our extensive experimental evaluations demonstrate that it improved the number of recognized gestures up to 2.4× more than the manually optimized baseline. The proposed technique can be extended in two directions. First, we aim to maximize the utility of wearable devices in general. Hence, this approach can be also applied to other applications whose accuracy can be characterized in a similar way, such as human activity recognition. Second, we currently use the available energy budget and accuracy targets as inputs. A holistic optimization could be achieved by adaptively choosing these targets at runtime as a function of the user task, environment, and backup energy level.

## Figures and Tables

**Table 1:** The major parameters.

Symbol	Description
$t_{g}$	Time spent by the device to infer a single gesture
$N_{g}\left(t_{g}\right)$	Number of gestures recognized in a finite horizon
$E_{g}\left(t_{g}\right)$	Active energy consumption of a single gesture
$E_{i}\left(t_{g}\right)$	Idle energy consumption of the device
$E_{b}$	Energy budget over a finite horizon
$E_{comm}$	Communication energy consumption of the device
$E_{act}^{\mu p}\left(t_{g}\right)$	Active energy consumption of the microcontroller
$E_{act}^{sen}\left(t_{g}\right)$	Active energy consumption of the sensor
$E_{idle}^{\mu p}\left(t_{g}\right)$	Idle energy consumption of the microcontroller
$E_{idle}^{sen}\left(t_{g}\right)$	Idle energy consumption of the sensor
$G_{acc}\left(t_{g}\right)$	Accuracy of gesture recognition

**Table 2:** The energy model parameters for different energy storage voltages.

Symbols	2.7 V		3.0 V		3.3 V	
	Baseline	Reduced	Baseline	Reduced	Baseline	Reduced
$E_{conn}$ (μJ)	30.8		31.8		30.9	
$P_{com}^{\mu p}$ (μW)	1134.4		1337.8		1584.7	
$P_{com}^{sen}$ (μW)	71.8		87.3		105.5	
$E_{pre}^{\mu p}$ (μJ)	86.8		89.2		92.1	
$E_{pre}^{sen}$ (μJ)	102.8		114.8		153.9	
$t_{conn}$ (ms)	66	200	66	200	66	200
$E_{post}^{\mu p}\left(t_{g}\right)$ (μJ)	207.4	158.1$\cdot t_{g}+$ 42.6	203.8	157.0$\cdot t_{g}+$ 48.2	212.5	171.1$\cdot t_{g}+$ 53.6
$E_{post}^{sen}\left(t_{g}\right)$ (μJ)	30.7	12.7$\cdot t_{g}+$ 15.7	37.6	24.7$\cdot t_{g}+$ 14.3	177.3	152.2$\cdot t_{g}+$ 33.8
$E_{acq}^{sen}\left(t_{g}\right)$ (μJ)	835.6	1047.0$\cdot t_{g}+$ 3.9	931.6	1157.0$\cdot t_{g}+$ 8.0	1040.0	1273.0$\cdot t_{g}+$ 18.3
